# “Accessory After the Factors”: A Rare Case of an Acquired Factor VIII Inhibitor in a 75-Year-Old Man on Rivaroxaban

**DOI:** 10.7759/cureus.18597

**Published:** 2021-10-08

**Authors:** Nikolas Gutierrez, James Park, Terrance Leighton

**Affiliations:** 1 Dermatology, 1st Marine Division, 1st Combat Engineer Battalion, Camp Pendleton, USA; 2 Family Medicine, Camp Pendleton, USA

**Keywords:** doac, acquired factor inhibitor, factor viii inhibitor, bethesda assay, factor xa inhibitor

## Abstract

Direct oral anticoagulants (DOACs) are used to treat several conditions such as non-valvular atrial fibrillation, deep vein thrombosis, and pulmonary embolism. DOACs and other anticoagulants block crucial steps in the coagulation cascade and ultimately prevent clot formation. Generally, individuals initiated on an anticoagulant are predisposed to or have a propensity to form clots. Patients with hemophilia are given anticoagulants only in very rare cases. In this report, we discuss the case of a 75-year-old man with a history of atrial fibrillation managed on rivaroxaban; he presented to the emergency department with fatigue, easy bleeding, symptomatic anemia, and significantly elevated partial thromboplastin time (PTT) with an undiagnosed acquired factor VIII inhibitor. Reports of DOAC use and concomitant factor inhibitor autoimmunization, as seen in this case, are scarcely explored in the existing literature. While DOACs are popular anticoagulants, their variable effects on both prothrombin time (PT) and PTT make it difficult to detect superimposed bleeding disorders. In patients with severe anemia or significant elevations in PT or PTT, an expedited workup, including factor assays, may be a reasonable option as evidenced by this case.

## Introduction

Acquired factor inhibitors develop through idiopathic autoimmunization against coagulation factors, which results in a rare bleeding disorder. Factor inhibitors often present clinically in the form of sudden onset of easy bleeding and bruising in elderly patients after a surgical procedure. The incidence of acquired factor inhibition increases with age and its diagnosis is often delayed due to the extensive laboratory analysis that is required to elucidate the cause. Current methods for the diagnosis of intrinsic factor inhibitors remain vulnerable to confounding factors in the presence of exogenous factor inhibition [1,2].

Direct oral anticoagulants (DOACs) are factor Xa antagonists that are widely used to treat anticoagulation in the elderly. They are often preferentially used in patients with conditions such as non-valvular atrial fibrillation as they do not require frequent drug monitoring. When assessing prothrombin time (PT) or partial thromboplastin time (PTT) in those on a DOAC, results may be within normal limits or slightly elevated for either value and are generally considered to be uninterpretable [3,4]. 

We present a rare case of an acquired factor VIII inhibitor presenting as fatigue, easy bleeding, symptomatic anemia, and significantly elevated PTT in a 75-year-old man on rivaroxaban. Furthermore, limitations of coagulation studies in those on DOACs are reviewed. Lastly, the role that current laboratory studies may play in delaying the diagnosis of superimposed bleeding disorders in those on anticoagulation therapy with a DOAC is discussed.

## Case presentation

A 75-year-old man with atrial fibrillation (with a CHA_2_DS_2_-VASc score of 3), chronic kidney disease, diabetes mellitus type II, and rheumatoid arthritis on rivaroxaban 15 milligrams per day presented to the emergency department with extreme fatigue and prolonged bleeding after a minor abrasion or laceration for the past one month. He also reported frank hematuria and two large ecchymoses on bilateral forearms. He denied any personal or family history of coagulation disorders.

His vital signs on initial presentation were notable for hypotension, tachycardia, and tachypnea; additionally, he was alert, oriented, and cooperative although he was in mild discomfort. His physical examination was notable for multiple, large ecchymoses on the upper and lower extremities. There was no appreciable gingival bleeding, petechia, or rectal bleeding; the remainder of his physical exam was unremarkable.

On review of his medical record, it was revealed that his primary care physician had noted, one week prior to the presentation to the emergency department, that he had been taking an old prescription (rivaroxaban 30 milligrams per day) in addition to his current dose (rivaroxaban 15 milligrams per day). This had resulted in him taking his DOAC at three times the prescribed dose following a recent decrease in dose due to his worsening kidney function. After the identification of his inappropriate dosing, he had stopped taking his rivaroxaban as instructed.

On initial presentation, complete blood count and coagulation panel were notable for hemoglobin of 4.8 g/dl (reference range: 13.5-17.5 g/dl) and significantly elevated PTT of 142 seconds (reference range: 25-35 seconds) despite not taking his rivaroxaban for one week; his platelet and white blood cell counts were within normal limits. Additionally, his comprehensive metabolic panel revealed a glomerular filtration rate of 35 mL/min (reference range: >50 mL/min) and his urinalysis with microscopy confirmed hematuria. The remainder of his laboratory studies, including a cardiac panel and a liver function test, were unremarkable.

He subsequently received two units of packed red blood cells for severe anemia and was admitted to the inpatient ward. Despite four additional days of observation while continuing to withhold his DOAC, he had persistent frank hematuria and bleeding from his peripheral intravenous sites. Additionally, his PTT failed to decrease as expected while holding his DOAC, and an expanded workup for a possible superimposed coagulopathy was initiated.

Mixing studies were obtained and were suggestive of factor inhibition. Factor activity assays were completed, and the patient was found to have an acquired factor VIII inhibitor. Following consultation with hematology, he was formally diagnosed with an acquired factor VIII inhibitor. 

The patient was treated with oral prednisone and recombinant porcine factor VIII; he was later discharged home after his bleeding and hematuria improved, with a plan for close outpatient monitoring. The patient's PTT was found to have normalized and his easy bleeding had resolved at his one-month follow-up appointment. He remained on prednisone for several months due to persistently elevated factor VIII inhibitor titer, and a slow taper was initiated once the levels decreased.

## Discussion

Acquired factor VIII inhibition, also known as acquired hemophilia A (AHA), is a rare cause of spontaneous bleeding that typically occurs in patients over the age of 50 years without any prior history of coagulopathy; a slight sex predilection favoring women over men has been described. Furthermore, associations with autoimmune and pro-inflammatory conditions have also been noted. This uncommon bleeding diathesis is classically suspected in a postoperative or peritraumatic period, which presents as large hematomas, considerable ecchymosis, and gingival bleeding. Albeit, unprovoked easy bleeding, ecchymosis, gingival bleeding, hematuria, and hemarthrosis may also be the presenting symptoms, thereby raising suspicion for the presence of an underlying factor inhibitor [1-3,5].

Factor VIII inhibitors are clinically suspected and diagnosed following laboratory evaluation with a coagulation panel, mixing studies, and factor assays. Coagulation tests may reveal an elevated PTT and normal PT; however, these findings are not specific to factor VIII inhibitors. The Bethesda assay, the preferred factor assay, is used to identify the specific factor inhibitor present and quantify the inhibitor titer via serial dilutions [1-3].

Treatment of this bleeding disorder typically involves desmopressin or recombinant factor VIII to stop any active bleeding and oral prednisone to decrease the activity of the factor VIII inhibitor. In addition to glucocorticoids, concurrent adjunct therapies such as cyclophosphamide, rituximab, and other immunosuppressant medications may be utilized. The duration of treatment is guided by the resolution of easy bleeding and the absence or decrease of inhibitor titer on serial factor assays [1-3].

Reports of DOAC use and superimposed factor inhibition, as seen in this case, are poorly represented in the existing literature: only one similar case has been previously published. It involves a 76-year-old woman on a DOAC for atrial fibrillation with severe abdominal pain, who was recommended for urgent surgery, complicated by prolonged PTT on initial laboratory studies despite withholding her DOAC. Her coagulation studies failed to normalize as anticipated based on half-life calculations, and additional coagulation studies were performed where she was found to have an acquired hemophilia with factor VIII inhibitors [5].

Similarly, our patient had persistent prolongation of his PTT that was initially attributed to his underlying chronic kidney disease. Moreover, given his use of supratherapeutic doses for weeks prior to the presentation, it was unclear as to when PTT normalization would occur in the setting of an elevated steady-state concentration and reduced renal function. Despite withholding his DOAC for several days, he had persistent bleeding, hematuria, and prolonged PTT; the Bethesda assay was completed, and acquired factor VIII inhibition was discovered.

In both cases, it is possible that anchoring on DOAC use as the sole cause of clotting dysfunction resulted in postponed evaluation to elucidate the cause of the patients' persistently prolonged PTT. Furthermore, it is possible that our patient’s DOAC use delayed the identification of his acquired hemophilia with factor VIII inhibitors, which may have led to his severe anemia and the need for blood transfusion; however, a direct association could not be established considering his history of supratherapeutic doses of his DOAC and diminished renal function. These two case reports illustrate how DOAC use may further complicate and possibly delay the identification of a superimposed coagulopathy.

While DOACs have unpredictable effects on PT and PTT, our patient’s marked elevation of PTT may have provided early evidence for superimposed hemophilia. In evaluations of DOAC use and the observed effect on coagulation values, it has been reported that PT and PTT elevations were rarely greater than one and a half times the upper limit of normal. Indeed, the elevations are often dose-dependent and, as was anticipated in the early course of our patient’s care, should normalize quickly following a decrease in dose or discontinuation [6-8].

Similarly, the discordance of a normal PT and a marked elevation of PTT was suggestive of an additional process at work. Due to his poor kidney function, this discrepancy was initially attributed to decreased renal clearance of rivaroxaban. While recommendations regarding the use of DOACs in chronic kidney disease are continuing to evolve, there is evidence to suggest that direct measurement of plasma DOAC levels should be available quickly enough to be clinically useful. Direct measurement of plasma rivaroxaban levels could have provided valuable elucidation of DOAC clearance in a patient for whom the theoretical half-life had limited validity [8,9].

DOACs may also interfere with coagulation factor assays. Specifically, the Bethesda assay, which is often the assay of choice to determine factor activity titers, and is dependent on PTT levels. While serial mixing with pooled plasma is meant to overcome factor inhibition, the presence of multiple factor inhibitors (such as a Xa inhibitor and a factor VIII inhibitor) makes it difficult to determine the titer of either inhibitor. For patients taking a DOAC with suspected superimposed coagulopathy, coagulation factor assays, such as the Bethesda assay, should be completed after exogenous factor inhibition has been discontinued and plasma drug concentration has declined [8].

Attempts to neutralize DOACs as part of laboratory protocols have not had much success, and factor assays should be viewed with caution in patients on a DOAC at the time of sample collection. This further underscores the limitations of laboratory testing that has not yet adapted to the need to account for a widely utilized class of anticoagulants whose misuse could present similarly to the intrinsic diseases these assays aim to identify. Until laboratory protocols evolve to provide more discernable results, for a physician managing a patient on a DOAC with a prolonged PTT, either significantly higher than or elevated for longer than expected, it is reasonable to consider an expanded workup for a superimposed coagulopathy initially and again after holding their DOAC if clinical suspicion persists [8].

Acute management of severe bleeding is aimed at correcting the coagulopathy resulting in disease. The treatment of rivaroxaban-associated bleeding includes fresh frozen plasma, recombinant factor Xa, and the recent Food and Drug Administration (FDA)-approved reversal agent andexanet alfa [10]. Aside from fresh frozen plasma, the therapeutic modalities specific for rivaroxaban-associated bleeding would have resulted in limited improvement in our patient’s severe disease as this would have only treated one component of his coagulopathy. As previously described, our patient did not improve until he received recombinant porcine factor VIII and oral prednisone, which was continued for several months after his discharge. Until laboratory protocol and testing improve enough to discern multifactor inhibition on initial presentation, acute treatment options for patients on anticoagulation therapy with a possible underlying coagulopathy will be limited. This delay may result in the need for more blood product transfusions, increased likelihood of transfusion-associated reactions, and prolonged hospitalization.

## Conclusions

While DOACs are a popular class of anticoagulants, their variable effects on both PT and PTT make it difficult to detect superimposed bleeding disorders. While de novo development of bleeding diatheses is rare, the significant number of patients who are on DOACs nationally suggests that several similar cases could occur each year. As noted in the context of this case, the development of more robust and accessible laboratory testing is called for given the limitations of current techniques when multifactor inhibition is suspected in the acute setting. These techniques rely on subtle constellations of findings and make it difficult to differentiate between disorders that have a large degree of overlap when evaluating initial coagulation assays and studies. In patients on anticoagulation therapy presenting with severe bleeding, symptomatic anemia requiring transfusion, or significant elevations in PTT to several times the upper limit of normal, an expedited workup, including factor assays, may be reasonably pursued at the time of presentation; initial laboratory results must be viewed with caution while exogenous factor inhibition is present. If clinical suspicion of an underlying coagulopathy persists despite indeterminant initial laboratory investigation, it may be reasonable to repeat coagulation studies and factor assays once anticoagulation therapy has been discontinued and serum drug levels decline.
